# Adapting and adopting highly specialized pediatric eating disorder treatment to virtual care: a protocol for an implementation study in the COVID-19 context

**DOI:** 10.1186/s43058-021-00143-8

**Published:** 2021-04-08

**Authors:** Jennifer Couturier, Danielle Pellegrini, Catherine Miller, Paul Agar, Cheryl Webb, Kristen Anderson, Melanie Barwick, Gina Dimitropoulos, Sheri Findlay, Melissa Kimber, Gail McVey, James Lock

**Affiliations:** 1 McMaster University, 1280 Main St W, Hamilton, Ontario Canada; 2 Canadian Mental Health Association – Waterloo Wellington, 1 Blue Springs Dr, Waterloo, Ontario Canada; 3 Chicago Center for Evidence-Based Treatment, 25 E Washington St, Chicago, Illinois USA; 4 Research Institute, The Hospital for Sick Children, 555 University Ave, Toronto, Ontario Canada; 5 University of Toronto, 155 College St, Toronto, Ontario Canada; 6 University of Calgary, 2500 University Dr NW, Calgary, Alberta Canada; 7 Research Institute, University Health Network, 200 Elizabeth St, Toronto, Ontario Canada; 8 Stanford University, 401 Quarry Rd, Stanford, California USA

**Keywords:** Implementation science, Eating disorders, Anorexia Nervosa, Virtual care, Family-based treatment, Fidelity, COVID-19, Children, Adolescents

## Abstract

**Background:**

The COVID-19 pandemic has negatively impacted individuals with eating disorders; resulting in increased symptoms, as well as feelings of isolation and anxiety. To conform with social distancing requirements, outpatient eating disorder treatment in Canada is being delivered virtually, but a lack of direction surrounding this change creates challenges for practitioners, patients, and families. As a result, there is an urgent need to not only adapt evidence-based care, including family-based treatment (FBT), to virtual formats, but to study its implementation in eating disorder programs. We propose to study the initial adaptation and adoption of virtual family-based treatment (vFBT) with the ultimate goal of improving access to services for youth with eating disorders.

**Methods:**

We will use a multi-site case study with a mixed method pre/post design to examine the impact of our implementation approach across four pediatric eating disorder programs. We will develop implementation teams at each site (consisting of therapists, medical practitioners, and program administrators), provide a remote training workshop on vFBT, and offer ongoing consultation during the initial implementation phase. Therapists will submit videorecordings of their first four vFBT sessions. We propose to study our implementation approach by examining (1) whether the key components of standard FBT are maintained in virtual delivery measured by therapist self-report, (2) fidelity to our vFBT model measured by expert fidelity rating of submitted videorecordings of the first four sessions of vFBT, (3) team and patient/family experiences with vFBT assessed with qualitative interviews, and (4) patient outcomes measured by weight and binge/purge frequency reported by therapists.

**Discussion:**

To our knowledge, this is the first study to evaluate an implementation strategy for virtually delivered FBT for eating disorders. Challenges to date include confirming site participation and obtaining ethics approval at all locations. This research is imperative to inform the delivery of vFBT in the COVID-19 context. It also has implications for delivery in a post-pandemic era where virtual services may be preferable to patients and families living in remote locations, where access to specialized services is extremely limited.

**Trial registration:**

ClinicalTrials.gov NCT04678843, registered on December 21, 2020.

**Supplementary Information:**

The online version contains supplementary material available at 10.1186/s43058-021-00143-8.

## Background

### COVID-19, mental health, and eating disorders

The COVID-19 pandemic has had a tremendously negative impact on mental health, including heightened anxiety, depression, and psychological distress in populations all over the world [1–4]. Literature on the pandemic’s impact on individuals with eating disorders (EDs) and their families is slowly emerging [5–8]. During preliminary lockdown measures and relative to before the pandemic, individuals with EDs across Spain, the USA, Netherlands, Australia, and the UK reported exacerbations of ED symptoms and behaviors, higher concerns of relapse, and reduced motivation for recovery, while also expressing greater concerns about COVID-19 on their mental health than their physical health [6, 9–11]. Specifically among children and early adolescents, a children’s hospital in Perth, Australia, reported a substantial increase in the number of children with Anorexia Nervosa requiring admission to hospital for nutritional rehabilitation over the course of the pandemic [12].

Worldwide closures of day hospitals are leaving the most severely ill individuals with EDs without life-saving treatment [6]. Across Canada, most clinical settings are permitting only urgent outpatient visits for medical reasons or admission to inpatient units, and in-person mental health ambulatory visits are reduced or suspended in many areas. As the ED population is particularly vulnerable and at significant risk of medical complications and death should they not receive care [13], there is an urgent need to prioritize the implementation of effective virtual treatment options.

### Evidence-based treatment and their adaptation

The most widely used and supported evidence-based treatment for children with EDs (in particular Anorexia Nervosa) is family-based treatment (FBT) [14–16]. Despite a growing body of evidence supporting the clinical effectiveness of FBT adapted for virtual care [17–20], challenges remain in its implementation in our current context. A recent consensus panel discussion conducted as part of a Canadian Institutes of Health Research (CIHR)-funded COVID-19 Knowledge Synthesis project, aimed at developing virtual care clinical practice guidelines, revealed uncertainty about the strength of evidence for virtual care, and noted a number of challenges related to the virtual implementation of FBT [21]. These include the conduct of the family meal, supervision of mealtimes, reliance on caregivers for obtaining accurate weights, involvement of siblings in the treatment process, and unexpected termination of sessions. As the timeline of a return to in-person delivery of outpatient mental health services is unknown, it is anticipated that virtual care will be the primary method of treatment delivery for the foreseeable future [22]; it is also possible that a blended model of virtual and in-person ED treatment may be more common post-pandemic. While this may present opportunities by improving access to service, the nature of virtual treatment adaptations and their effective delivery requires examination to ensure patient outcomes do not suffer. Adaptations that focus on improving treatment fit with the target population can lead to improved engagement, acceptability, and clinical outcomes [23], but modifications that alter or remove core components of a treatment, or fail to align with population needs, may be less effective [24]. Failing to understand the type of modifications that occur and the systematic processes that lead to successful virtual treatment implementation within a given ED program may hinder outcomes [25, 26].

### Our previous implementation research

Our team recently completed research on the implementation of standard in-person FBT in Ontario within our pre-existing network of ED care providers [27–29]. We examined FBT implementation in four pediatric ED programs in Ontario [28]. Our implementation approach, informed by the Active Implementation Framework (AIF) [30], the Consolidated Framework for Implementation Research (CIFR) [31], and the Implementation Outcomes Taxonomy (IO) [32] included (1) the development of implementation teams at each site, (2) provision of FBT training, (3) monthly clinical and implementation consultation, and (4) fidelity assessment. Implementation effectiveness was assessed using mixed methods. We found that fidelity scores were similar to those in studies from academic centres [28]. Participants perceived our implementation approach as facilitating adoption of FBT in their organizations, and they were delivering standard FBT with fidelity to the key components of the model [28]. They reported that involving program administrators and medical practitioners (Doctor of Medicine [MD] or Nurse Practitioner [NP]) in implementation processes was essential to ensuring implementation success [27, 28]. Remaining highly engaged, the organizations requested ongoing consultation beyond the study time parameters [27–29].

### Study purpose and rationale

In light of the challenges caused by the COVID-19 pandemic and the evidence of clinical and implementation effectiveness of our approach [28], there is an urgent need and opportunity to adapt FBT for virtual delivery for adoption across our network. As noted above, the adaptations required for virtual delivery of FBT require examination to ensure maintenance of the key components and fidelity to the treatment. Therefore, we propose to study the initial implementation of virtual FBT (vFBT) in four pediatric programs in Ontario, with goals of building on our previous work, further developing clinical capacity for virtual care within our system, and improving access to evidence-based treatment for children with EDs in the COVID-19 context and beyond.

### Overview of our proposed research

We propose to study the initial adaptation and adoption of vFBT using the Quality Implementation Framework (QIF) [33] to guide our implementation process. The QIF provides a 14-step process to guide activities in four phases of implementation. This implementation process has been successfully applied in our own research in ED programs [28] and in research by team members with other child and youth mental health organizations [34]. The Implementation Outcomes Taxonomy (IO) [32] will guide evaluation of implementation outcomes. The Standards for Reporting Framework for Implementation Studies [35] (STaRI; co-authored by MB) will guide our methodology and reporting (see Additional file 1). The Standard Protocol Items: Recommendations for Interventional Trials (SPIRIT) checklist [36] will guide the evaluation of our study design (see Additional file 2).

### Research questions

1. Does our adaptation to vFBT maintain the key components of standard FBT by therapist self-report?
2. Does our implementation approach for vFBT lead to implementation success at the participating sites, as measured by expert rating of fidelity to the vFBT model?
3. What is the experience of implementation teams and patients/families implementing vFBT (and for therapists, does the experience change over time)?
4. Do patients in vFBT demonstrate clinical outcomes similar to patients receiving standard FBT?

## Methods/design

This study is funded by the Canadian Institutes of Health Research (CIHR). The protocol has received ethics approval from the Hamilton Integrated Research Ethics Board (HiREB), the St. Joseph’s Care Group Research Ethics Board, the North York General Hospital Research Ethics Board, and the Southlake Regional Health Centre Research Ethics Board. The fourth participating site, Canadian Mental Health Association – Waterloo Wellington, does not have an internal ethics review board, but the protocol was approved by their Quality and Risk, and Privacy departments.

### Virtual study communication and data collection

All communication and data collection will be done virtually using Zoom Healthcare (HIPAA/PHIPA-complaint videoconferencing software), telephone, e-mail, online surveys (Qualtrics), and SignNow. This will be done to mitigate any in-person contact due to COVID-19 social distancing regulations, as well as to reduce barriers to participation related to geography.

### Study design

We will use a multi-site case study with a mixed method pre/post design [37] to examine our implementation approach. We will collect quantitative measures evaluating therapist adherence to key components of FBT, therapist fidelity to the vFBT model, therapist change in readiness, attitudes, and confidence in implementing vFBT over time, and patient outcomes (changes in weight and binge/purge frequency). Qualitative data will be collected using virtual focus groups and interviews to understand implementation team and patient/family experiences of vFBT.

### Setting

We will work with four pediatric ED programs affiliated with the Ontario Community Outreach Program for Eating Disorders (OCOPED; led by GM), who provide psychotherapeutic intervention to children and adolescents under the age of 18 years. These four participating organizations (from a potential 22 organizations within OCOPED) voiced their interest, readiness, and capacity to implement vFBT in our preparatory work for this project.

### Eligibility criteria

#### Therapists, medical practitioners, and program administrators

To participate in this study, these individuals must work in Ontario, Canada, have the ability to read, understand, speak, and write in the English language and have access to a working computer/electronic device with a stable internet connection. Specifically, therapists must have prior training and supervision in standard FBT and be actively delivering FBT within their treatment programs. Medical practitioners must either be doctors (MD) or nurse practitioners (NP). Program administrators must currently manage an ED program.

#### Patients

To participate in this study, patients must be under 18 years of age; have a diagnosis of Anorexia Nervosa; live in Ontario, Canada; have the ability to read, understand, speak, and write in the English language; and have access to a working computer/electronic device with a stable Internet connection.

### Sample size

The four ED programs participating in the study average two therapists per program, yielding an approximate sample size of eight therapists, four medical practitioners, and four program administrators. We are aiming to recruit one family with an eligible patient per therapist at each site (eight families total). Although a sample size calculation was not completed, we believe this is an appropriate sample size for a feasibility study with this population.

### Procedures and timelines

#### Completed previously: phase 1—initial considerations regarding the host setting

Our implementation approach will follow the stages identified by the QIF [33]. Phase 1 site engagement and buy-in has been completed, by virtue of a well-established network that worked together on previous implementation studies. Of the four ED programs that voiced interest and capacity in implementing vFBT, two programs participated in our prior implementation study. The other two sites have subsequently received training in standard FBT through OCOPED training events. Also, during this time (pre-funding), we identified (with vFBT model co-developer JL) the types of adaptations required for virtual delivery in vFBT to ensure they do retain the key components of the standard FBT model, as these are essential for treatment effectiveness. These adaptations are summarized with the Framework for Reporting Adaptations and Modifications – Enhanced (FRAME) [38] in mind in Table 1.

**Table 1:** Adaptations to family-based treatment (FBT) for virtual delivery

Component of FBT	What is the adaptation?	Why was the adaptation made?	Was the adaptation planned or unplanned?	What are the implications for fidelity?
**Weighing**	Therapist weighs patient virtually with patient at home instead of in clinic (home scale is required).	Patient is unable to come into the clinic for in-person visits.	Planned (proactive adaptation).	The patient’s weight is captured as intended. The process of weighing is maintained.
**Time spent alone with adolescent**	Parents are asked to leave the room for the first 10 min of the session so that individual time can be spent between the adolescent and therapist.	In a clinic setting, the adolescent is taken from the waiting room. In virtual therapy, family members often come on screen together, so parents must be asked gently and respectfully to leave.	Planned (proactive adaptation).	Time spent alone with adolescent is maintained.
**Sharing the weight graph**	Therapist plots the weight graph and shares it over the screen by holding up the sheet of paper. Weight graph is reviewed together virtually. The graph could also be plotted electronically by therapist and they could use the “share screen” feature.	Usually the graph is easily shared in person. Virtually, it can be difficult for the family to see the graph on paper; therefore, using the “share screen” feature can be helpful.	Planned (proactive adaptation).	The weight graph is shared and reviewed as intended.
**Family meal**	Family meal (observed by the therapist) is completed virtually, with the ability of parents to gather additional food items if needed (since they are in their own homes).	When done in person, the option of additional food items is limited. With the family at home they have many more options available to them.	Planned (proactive adaptation).	Family meal is conducted as intended.
**Therapeutic principles**	All sessions are to be completed virtually and with the same principles as in-person treatment with emphasis on externalization and agnosticism. Externalization can be depicted by drawing on the “white board” feature of the virtual platform.	Drawing on a white board or piece of paper is typically done in clinic to depict externalization. This can be done on some platforms with the “white board” and/or share screen feature.	Planned (proactive adaption).	Therapeutic principles are maintained in virtual delivery.

#### Months 0–4: phase 2: creating a structure for implementation: formation of implementation teams, vFBT training, training evaluation, and full-implementation preparation

Implementation teams will be developed at each site and include program staff who are knowledgeable about the FBT model, as well as organizational processes and procedures affecting implementation. These teams will be responsible for implementing vFBT, and their leadership at the organizational level is critical for effectiveness and sustainability [27–29]. Based on our prior work, implementation teams will include a therapist, a medical practitioner, and a program administrator [28]. The Lead Principal Investigator (LPI) (JC) will meet by Zoom Healthcare with all sites to formalize implementation team membership and discuss the implementation process. Implementation teams will meet with the research team (LPI, research coordinator, and research assistant) by Zoom Healthcare on a bi-weekly basis to monitor the implementation process, discuss any issues with vFBT delivery or sustainment, and identify any further vFBT adaptations that may be necessary. Implementation teams will also be encouraged to meet on a weekly basis independent of the research team, to develop their internal capacity to manage implementation activities.

Ethics approval will be sought from each organization during this phase. Participants will be consented by our research team remotely. All therapists, medical practitioners, and program administrators will undergo vFBT training via Zoom Healthcare over a half day period to review vFBT implementation, key components of FBT to be maintained, fidelity to the vFBT model, potential barriers to success, and experiences to date. Training will be led by external experts (JL, KA) and local experts (JC, CW) who have been delivering vFBT for over 1 year related to another study (led by JL). A section of training will be devoted to how medical practitioners, and program administrators can support the implementation of vFBT within their respective roles (led by SF and PA, respectively).

#### Months 5–8: phase 3: ongoing structure once implementation begins: implementation of vFBT in practice

Phase 3 of implementation is the period during which learning becomes integrated into practitioner and program practices and therapists begin delivery of vFBT with concomitant fidelity assessment. Implementation team members will continue to participate in bi-weekly Zoom Healthcare meetings with the research team and meet weekly independent of the research team. Throughout Phase 3, therapists will recruit patients/families (with the research team obtaining consent) and will submit Zoom Healthcare videorecordings of vFBT sessions 1, 2, 3, and 4 to the research team using a secure cloud-based platform (OpenText/Hightail) for transferring large files. These sessions will be rated for fidelity by an expert in fidelity rating (JC, GD). Therapists will participate in bi-weekly group clinical consultation meetings led by vFBT Trainer LPI (JC) by Zoom Healthcare, to troubleshoot general clinical vFBT issues. Therapists at each site will be encouraged to meet independently from the research team for peer supervision on a weekly basis.

#### Months 9–12: phase 4: improving future applications: evaluating the experience of the implementation intervention

The evaluation phase of our implementation approach will involve the solicitation of feedback about the overall implementation process and a consideration of any further adaptations to the vFBT model that may be necessary for effective and sustained use over time. Implementation teams will continue to meet on a weekly basis without the research team, and therapists will continue to meet on a weekly basis for peer supervision, without the research team. One focus group will be completed at each organization, involving the participating therapists, medical practitioner, and program administrator. Focus group questions will elicit user experiences of the implementation process, perceptions about implementation success, and areas needing further adaptation. Semi-structured qualitative interviews will also be completed with patients and their families to elicit their perceptions of virtual treatment. Quantitative measures will also be evaluated, as described below.

### Measures

#### Key components of FBT (question 1)

Each therapist will submit a self-report questionnaire entitled the Key Measures of Therapist Behaviours and Self-Efficacy in FBT (developed by JL) as an indicator of their adherence to the key components of standard FBT within the vFBT model. This questionnaire will be completed after session 4 of vFBT and will be submitted using an online survey tool (Qualtrics).

#### Fidelity to the vFBT model (question 2)

Each therapist will videorecord vFBT sessions 1, 2, 3, and 4 with one patient and their family on Zoom Healthcare and submit these recorded sessions to the LPI via a secure file sharing platform for transferring large files (OpenText/Hightail). Experts in FBT fidelity rating (JC, GD) will rate the recordings using the FBT Fidelity and Adherence Check (FBT-FACT) [39, 40].

#### Implementation team and patient/family experiences of vFBT (question 3)

The experiences of therapists, medical practitioners, and program administrators implementing vFBT across settings will be evaluated qualitatively using the method of fundamental qualitative description [41]. We will ask participants at each site to undergo a semi-structured focus group with a member of our research team which will be recorded using Zoom Healthcare. These qualitative evaluations will focus on the execution of the implementation process, and the overall perceived success of the implementation. Patients/families will also participate in a semi-structured qualitative interview to share their experience of vFBT. A similar process of videorecording, transcription, and analysis will occur for focus groups and for family interviews.

In addition to these qualitative methods, quantitative measures will be collected from therapists to examine their change in readiness, attitudes, and confidence for virtual treatment delivery over time. Therapist individual readiness for change will be evaluated using the Brief Individual Readiness for Change Scale (BIRCS) [42], their attitudes about evidence-based practice will be assessed using the Evidence Based Practice Attitudes Scale (EBPAS) [43], and confidence related to the intervention will be assessed by administering an adapted version of the Perceived Attributes of the Principles of Effectiveness Scale (MPAS) [44]. These measures will be delivered at implementation baseline (at the time of consent), immediately following the vFBT training workshop, and after session 4 of vFBT has been completed (all by Qualtrics). These measures will help us evaluate any change in readiness, attitudes, and confidence over time so that we can better understand therapist experiences with the implementation approach. Therapist, medical practitioner, and program administrator demographics will also be collected at implementation baseline (at the time of consent).

#### Patient outcomes (question 4)

Patient outcomes including weight and number of binge/purge episodes per week will be recorded at treatment baseline (at the time of patient consent) and then submitted on a weekly basis after each of the first four vFBT sessions by the therapist (using Qualtrics). Weight gain of at least 2 kg following the first four sessions of FBT is highly predictive of final treatment outcome [45, 46] and is a valid measure of overall clinical effectiveness. This will be our benchmark for comparing vFBT effectiveness to standard FBT. Patient demographics and diagnosis will be collected at treatment baseline (at the time of patient consent).

### Analysis

#### Key components of FBT (question 1)

The Key Measures of Therapist Behaviours and Self-Efficacy in FBT will be examined to determine the percentage of therapists reporting that they completed all key components of FBT (weighing their patient, using externalization, using agnosticism, and doing a family meal in session 2 within vFBT).

#### Fidelity to the vFBT model (question 2)

The percentage of therapists demonstrating vFBT fidelity will be determined by the FBT fidelity rating scale (FBT-FACT [39]) rated by experts and achieving a 4/7 average score or greater on each session [40].

#### Implementation team and patient/family experiences of vFBT (question 3)

Transcripts from the end of study focus groups with teams and patients/families will be analyzed using conventional content analysis and the constant comparison technique [47]. Specifically, iterative reviews of text within and across transcripts will be completed by two members of the research team to identify codes and associated categories characterizing implementation team and patient/family experience with vFBT, their suggestions for change (if any), and to provide a rich description of their perceptions of the value and impact of the vFBT model. Codes and categories will be grouped into overarching themes that help to synthesize the qualitative data and address our research questions. In terms of quantitative measures, changes over time in readiness for change, attitudes toward evidence-based practice, and confidence related to vFBT will be analyzed using repeated measures ANOVA (using SPSS software). We will also examine demographic characteristics of therapists, medical practitioners, and program administrators including age, gender, academic discipline/area of expertise, and number of years in their current role, in relation to implementation success.

#### Patient outcomes (question 4)

As mentioned above, therapists will provide weekly data on patients’ weight and number of binge/purge episodes per week before starting vFBT and after each of the first four sessions of vFBT (using Qualtrics). Changes in weight and average number of binge/purge episodes per week over time will be analyzed using repeated measures ANOVA (using SPSS software). Weight gain will be compared against the benchmark of 2 kg gain after four sessions in standard FBT as a measure of clinical implementation success. Patient demographics, including age, gender, and number of years with the ED, will be examined in relation to outcome.

### Trial status

At the time of manuscript submission, we are in the process of obtaining consent to participate in this study from all therapists, medical practitioners, and program administrators and are preparing for the vFBT training workshop. No data cleaning or analysis has occurred.

## Discussion

As outlined in this protocol, this implementation study will evaluate the processes involved in adapting and adopting vFBT to treat children and adolescents (<18 years) with Anorexia Nervosa. To examine the impact of our implementation approach, we will be using a multi-site case study with a mixed method pre/post design involving four Ontario-based pediatric ED programs. Site implementation teams have been established and consist of therapists, medical practitioners, and program administrators, who will all participate in a remote vFBT training workshop and receive ongoing support and consultation for the duration of the trial. Qualitative and quantitative measures will be used to measure: (1) vFBT maintenance of the key components of standard FBT by therapist self-report, (2) fidelity to the vFBT model by expert rating, (3) participant experiences with vFBT, and (4) patient outcomes. Using the findings of this pilot study, our long-term goal is to expand access to vFBT for all treatment-seeking children and adolescents with Anorexia Nervosa and other EDs.

The results of this study can be used to inform future implementation of vFBT across Ontario and even throughout Canada. The effectiveness of our implementation approach will provide validation of the QIF framework generally and within the context of guiding implementation of vFBT in other ED programs. Adaptations from standard FBT to vFBT and demonstrating fidelity to key components in this mode of delivery will further our understanding of the fundamental mechanisms required to effectively deliver virtual care in this field. Provision of training and supervision in vFBT will increase the capacity of therapists to deliver FBT virtually within Ontario’s ED network—so crucial as a viable option for outpatient care during the social distancing requirements brought on by the COVID-19 pandemic [48].

The study will be completely virtual, with no in-person contact, including delivery of the vFBT training workshop, focus groups and interviews, and participant questionnaires/measures. As our previous FBT implementation study had an in-person training workshop, advantages and disadvantages were noted. Advantages included enhanced social connection, freedom from work duties, the opportunity to travel to a new city, and professional networking. Disadvantages included implementation team members having to travel to one location to partake in the training workshop, which could impede trial participation due to factors of cost, time, travel duration, and inconvenience. Additionally, the investigators also travelled to each site, which was costly and time intensive given the site locations across Ontario [28]. In the present trial, the four participating sites are all located within Ontario, Canada, eliminating issues of time zone differences, although two of our co-investigators who will be leading the virtual workshop are located in the USA, and as such, time zones will only need to be considered during the workshop portion of this study.

At the time of submission of this protocol, we received ethics approval from all sites involved; however, this process has been challenging and took much longer than originally expected. Given the high volume of COVID-19-related research projects, ethics approval at our institution took several months to obtain. Ethics procedures for our four participating sites had to be examined and followed as well. This process took several months, as all sites had different application requirements, as well as differing research ethics board meeting dates and deadlines. Each of the research ethics boards expressed their own unique concerns to be addressed. Approval from the other sites also had to be submitted back to our own board. Delays in approval had already been apparent, with staff on ethics boards being redeployed to aid with COVID-19-related efforts, and the large abundance of research that is being conducted at each site. Issues with ED-related research delays and interruptions have been common during the pandemic, as described in the literature [49]. We will monitor any impact that these delays may have on our trial.

The increased burden on healthcare services and associated clinical teams during COVID-19 [50] has already impacted our study preparations. Initially, we proposed to study six sites; however, two sites declined to participate due to higher-than-normal clinical caseloads and decreased staffing resulting from the COVID-19 pandemic. We have been unable to recruit replacement sites despite approaching seven other ED programs across Ontario.

We anticipate additional pandemic-related challenges during this study, but novel benefits of virtual care could also be revealed. Increased caregiver burden has been reported during telehealth visits, with accounts of parents having to weigh their child at home [8, 17], as well as additional burdens of supporting virtual school at home. However, increased time spent at home during COVID-19 has had positive effects on some families with EDs, such as building greater connections with family members and more time for self-care [9]. The second treatment session in FBT involves a family meal, with the therapist present and observing the family. While this is feasible during in-person treatment, therapists and families may find this challenging to complete virtually. However, there could be additional benefits to the family being within their own home during the observed family meal. This may present an opportunity to increase an insufficient meal due to the family being at home and able to supplement with food they already have. Technological challenges may reveal caregiver, patient, and/or therapist preferences for in-person rather than virtual treatment, although advantages to virtual care may also emerge, such as improved access to care for those located in remote areas, or greater convenience for families (i.e., no parking fees, no travel time, reduced need/cost for childcare).

At the time of this writing, COVID-19 cases are increasing exponentially, and health care workers are experiencing fatigue, and some are falling ill. As such, hospital departments may become even more understaffed and overworked. With ED programs across Ontario seeing more than three times the typical number of referrals for outpatient care, and an unprecedented number of children requiring inpatient hospitalization, therapists, medical practitioners, and program administrators participating in our study may no longer have time to commit to implementation activities and the tension for change may shift to more pressing demands for the time being. As there is much uncertainty surrounding the recession of the COVID-19 pandemic, it is nearly impossible to fully anticipate the impact it will have on those conducting research, implementing new treatment modalities, and providing clinical care.

In summary, this is the first clinical trial to evaluate the implementation of vFBT in pediatric EDs, specifically with children and adolescents with Anorexia Nervosa. Challenges to date include obtaining ethics approval at participating sites and difficulties in recruiting additional sites to join our study. Results from this trial have the potential to provide valuable information about adaption from in-person to virtual care and how best to implement vFBT in pediatric ED treatment in Ontario, which will be useful when considering treatment for those in remote areas where in-person specialized care may be unavailable or inaccessible. In addition, this research will help to inform further studies on how the virtual delivery of FBT compares with in-person “gold standard” models. Future studies could explore the costs associated with the implementation and virtual delivery of vFBT in comparison to standard, in-person FBT.

## Supplementary Information

**Additional file 1.** A guide for the testing of an implementation intervention.

**Additional file 2.** A guide for evaluating the study design.

## Data Availability

Not applicable.

## Abbreviations

AIF: Active Implementation Framework
BIRCS: Brief Individual Readiness for Change Scale
CIFR: Consolidated Framework for Implementation Research
CIHR: Canadian Institutes of Health Research
EBPAS: Evidence-Based Practice Attitudes Scale
ED: Eating disorder
FBT: Family-based treatment
FBT-FACT: Family-Based Treatment Fidelity Adherence and Check
FRAME: Framework for Reporting Adaptations and Modifications - Enhanced
HiREB: Hamilton Integrated Research Ethics Board
IO: Implementation Outcomes Taxonomy
LPI: Lead Principal Investigator
MD: Doctor of Medicine
MPAS: Perceived Attributes of the Principles of Effectiveness Scale
NP: Nurse Practitioner
OCOPED: Ontario Community Outreach Program for Eating Disorders
QIF: Quality Implementation Framework
SPIRIT: Standard Protocol Items: Recommendations for Interventional Trials
STaRI: Standards for Reporting Framework for Implementation Studies
vFBT: Virtual Family-Based Treatment
